# Harnessing the Potential of Plant Expression System towards the Production of Vaccines for the Prevention of Human Papillomavirus and Cervical Cancer

**DOI:** 10.3390/vaccines10122064

**Published:** 2022-12-01

**Authors:** Balamurugan Shanmugaraj, Ashwini Malla, Christine Joy I. Bulaon, Waranyoo Phoolcharoen, Natacha Phoolcharoen

**Affiliations:** 1 Baiya Phytopharm Co., Ltd., Bangkok 10330, Thailand; 2 Center of Excellence in Plant-Produced Pharmaceuticals, Chulalongkorn University, Bangkok 10330, Thailand; 3 Department of Pharmacognosy and Pharmaceutical Botany, Faculty of Pharmaceutical Sciences, Chulalongkorn University, Bangkok 10330, Thailand; 4 Department of Obstetrics and Gynecology, Faculty of Medicine, Chulalongkorn University, Bangkok 10330, Thailand

**Keywords:** cervical cancer, human papillomavirus, recombinant drugs, immunotherapy, therapeutic vaccine, plant-made pharmaceuticals

## Abstract

Cervical cancer is the most common gynecological malignant tumor worldwide, and it remains a major health problem among women, especially in developing countries. Despite the significant research efforts employed for tumor prevention, cervical cancer ranks as the leading cause of cancer death. Human papillomavirus (HPV) is the most important risk factor for cervical cancer. Cervical cancer is a preventable disease, for which early detection could increase survival rates. Immunotherapies represent a promising approach in the treatment of cancer, and several potential candidates are in clinical trials, while some are available in the market. However, equal access to available HPV vaccines is limited due to their high cost, which remains a global challenge for cervical cancer prevention. The implementation of screening programs, disease control systems, and medical advancement in developed countries reduce the serious complications associated with the disease somewhat; however, the incidence and prevalence of cervical cancer in low-income and middle-income countries continues to gradually increase, making it the leading cause of mortality, largely due to the unaffordable and inaccessible anti-cancer therapeutic options. In recent years, plants have been considered as a cost-effective production system for the development of vaccines, therapeutics, and other biopharmaceuticals. Several proof-of-concept studies showed the possibility of producing recombinant biopharmaceuticals for cancer immunotherapy in a plant platform. This review summarizes the current knowledge and therapeutic options for the prevention of cervical cancer and discusses the potential of the plant expression platform to produce affordable HPV vaccines.

## 1. Introduction

Cancer, one of the most prevalent fatal diseases, is characterized by uncontrolled cell growth, resulting in the dissemination of cancerous cells from one part of the body to other parts (i.e., secondary tumors) [1]. Significant developments in our understanding of cancer mechanisms and the advancement of modern medicines in the last decade has transformed the life of many cancer patients. However, cancer treatments are expensive and the cost of long-term treatment continually increases annually. Eventually, the economic burden caused by cancer treatments due to the huge expenses puts extreme burdens on patients and their families [2]. Thus, the increasing incidence and prevalence of cancer imposes serious socioeconomic burdens, especially in resource-limited nations; hence, it is highly essential to find an alternative therapeutic option [3].

Cervical cancer occurs on the surface of the cervix, which is the narrow opening into the uterus connecting the vagina. Squamous cell carcinoma and adenocarcinoma are the two sub-types, which originate from squamous cells in the ectocervix and glandular cells in the endocervix, respectively. Squamous cell carcinoma accounts for 75% of cervical carcinoma cases [4]. An estimated 569,847 new cases and 311,365 deaths occurred in 2018 [5,6]. More than 85% of these cases occur in low- and middle-income countries (LMICs), where cervical cancer is the leading cause of cancer among women, and the death rate is 18 times higher than in high-income countries [4]. The mortality associated with cervical cancer is expected to increase to approximately 410,000 by 2030 [7,8]. The global disparities in mortality and the incidence of cervical cancer are due to a lack of organized screening and prevention programs, including treatment of women who have pre-cancerous lesions [9]. Clinical capacity is also an issue in many low-resource areas. As a result, effective screening services might be unavailable, leading to patients presenting with advanced-stage cervical cancer with limited and unaffordable treatment options in many LMICs.

Common types of medical intervention for cervical cancer include surgery, radiation, chemotherapy, targeted drug therapy and immunotherapy. Surgery is the generally preferred option for cervical pre-cancers and invasive cervical cancers [10]. Chemotherapy as another option involves administering anti-cancer drugs either orally or intravenously. Though chemotherapy is considered the most effective mode of treatment, it involves aftereffects such as nausea, vomiting, loss of appetite, hair loss, body sores, fatigue, and nerve and kidney damage, which vary based on the type of drug, dosage, and exposure length [11].

Cervical cancer is a preventable disease and key preventive initiatives include screening, vaccination, and educating about the risk factors. It is a hard fact that, although preventable, it is the leading cause of cancer deaths in developing nations. In 2018, Dr. Tedros Ghebreyesus, the Director General of the World Health Organization (WHO), called for global action towards the elimination of cervical cancer, and in August 2020, the World Health Assembly adopted a global strategy to eliminate the same. The key goals to reach global targets by 2030 to get on the way to eliminating cervical cancer within the next century are vaccination (90% vaccination coverage of girls at 15 years of age), screening (70% coverage of screening twice in a lifetime), and treatment (90% treatment and care). Based on this WHO triple-intervention strategy, 90-70-90 targets should be met by each country by 2030. Implementing this strategy will save more than 62 million women’s lives over the course of the next century. The WHO Cervical Cancer Elimination Modelling Consortium (CCEMC) predicted the impact of screening, HPV vaccination, and treatment strategies on cervical cancer in 78 LMICs. Over the next century, the triple-intervention strategy could avert 14.6 million deaths in 2070 and 62.6 million deaths in 2120 with vaccination and twice-lifetime screening [12].

## 2. Human Papillomavirus (HPV) and Cervical Cancer

HPV is a non-enveloped, double-stranded, DNA virus belonging to the family *Papillomaviridae*. It contains 8 kb circular genome encoding for early region and late region proteins, such as E1, E2, E4, E5, E6, E7, E8, L1, and L2, which are necessary for viral replication and capsid proteins [13]. There is strong evidence linking high-risk HPV to cervical cancer as a cause. Almost all cervical cancer is caused by persistent infection with high-risk HPV, in which HPV-DNA is detected in 95% to 100% of cervical cancer specimens worldwide [14]. The DNA of high-risk HPV integrates into the host cell genome and causes neoplastic cellular changes. Cervical, vulvar, vaginal, penile, and anal neoplasia lesions can be caused by HPV infection. The junction of columnar epithelium representing the endocervix and squamous epithelium representing the exocervix are the more susceptible zones for the infection. Among several types of HPV, 15 high-risk genotypes are linked to cervical neoplasia. HPV is the most common sexually transmitted infection. Almost 70% of invasive cervical cancer cases were caused by HPV-16 and HPV-18, of which HPV-16 alone contributes to 54% of the total cases [15]. The average lifetime probability of acquiring HPV is 85% and 91% among women and men, respectively, who have had at least one sexual partner [16].

The International Federation of Gynecology and Obstetrics categorized stages 0 to IV for cervical cancer [17]. The risk and progression factors that increase the chance of cervical cancer include an active sexual life at an early age; multiple sexual partners; tobacco use; high parity, long-term use of oral contraceptives; Chlamydia or HIV co-infection; and malnutrition or poor diet [18]. Primary preventive measures include following safe sexual health practices, heeding warnings about tobacco use, early detection, and vaccination. In addition, regardless of vaccination status, frequent cervical cancer screening is highly recommended. Approximately 80% of individuals with HPV will clear the infection spontaneously within 18 to 24 months of infection [19]. In most women, the HPV infection is not persistent, the virus is cleared naturally, and the dysplasia regresses. In a small percentage of women, the infection is persistent, and the transformed cells can replicate and progress to cancer after several years [20,21,22]. In women with persistent HPV infection, 3–5% will develop the significant pre-invasive disease, and less than 1% will develop cervical cancer [23].

## 3. Cervical Cancer Vaccines

Three HPV vaccines have been successfully developed and approved for commercial use until now (Table 1). These virus-like particles (VLPs) vaccines are based on the HPV L1 capsid protein, which self-assemble in the recombinant expression system. The first FDA approved HPV vaccine, Gardasil (Merck and Co, Kenilworth, NJ, USA), a quadrivalent vaccine made of capsid proteins from HPV types 6, 11, 16, and 18, is the first commercially available HPV vaccine. Another vaccine, Cervarix (GlaxoSmithKline Biologicals, Rixensart, Belgium), is a bivalent HPV vaccine that contains capsid proteins from HPV types 16 and 18, along with monophosphoryl lipid A (MPL) and aluminum hydroxide as an adjuvant. Furthermore, a nonavalent vaccine, Gardasil 9 (Merck and Co, Kenilworth, NJ, USA), was approved by the Food and Drug Administration (FDA); it provides additional protection from HPV types 31, 33, 45, 52, and 58, which cause 20% of cervical cancers. Gardasil and nonavalent vaccines use aluminum hydroxide as an adjuvant. A three-shot regimen of intramuscular injections of these vaccines has shown better efficacy in decreasing HPV and HPV-related infection rates worldwide [24]. The HPV-vaccination gap exists between the different countries; hence, the implementation of the HPV vaccine is urgently required for public health intervention. For instance, 32% of adolescent females in high-income countries received the full course of HPV vaccines, whereas only about 1% received HPV vaccines in LMICs [25].

## 4. Treatment Strategies

Significant advancements in treating gynecological malignancies have been achieved in recent years. Primary treatment options for early-stage cervical cancer include surgery or radiation therapy, with or without chemotherapy. For small precancerous lesions and stage I cancer, surgery is usually the standard core of treatment. The five-year survival rate is estimated at 65–95% in the early stage. The choice of therapy depends on the stage of cancer, patient co-morbidities, and risk factors for recurrence, and each treatment measures has its own limitations [26]. Unfortunately, many patients, especially in LMICs, are diagnosed with an advanced stage that worsens the prognosis [23]. The management of metastatic or recurrent cervical cancer depends on the extent of the disease. For patients with a limited metastatic lesion, treatment can be focused on the area of recurrence using radiation therapy or a surgical approach in selected cases. However, chemotherapy is the preferred treatment option for women with a more extensive disease condition.

Immunotherapy was revealed as another attractive approach with impressive results in other solid malignancies, such as melanoma and lung cancer. Several significant immunosuppressive parameters have been revealed that support the promise of immunotherapy in cervical cancer. The tumor microenvironment invaded the immune responses and surveillance of immune cells (macrophages, monocytes, dendritic cells, natural killer cells, antigen presenting cells, B- and T-lymphocytes) in the human body, vanquishing their functioning by various mechanistic modes depending on their vicinity [27,28,29,30]. Immunotherapy has revolutionized the tumor treatment strategy with the advent of immune check point proteins that act as a gateway of immune responses with inhibitory or stimulatory signals in the immune cells [31,32,33]. Programmed cell death-1/PD ligand-1 (PD-1/PD-L1) inhibitors could be a cutting-edge strategy that has achieved significant efficacy results in many cancer types and improved the clinical outcomes in cancer patients. The PD-L1 was presented on the cancer cell surface, tumor infiltrating lymphocytes, antigen-presenting cells, and PD-1 was expressed on T cells. HPV infection is correlated with increased PD-L1 expression *in vivo* [34]. Additionally, high PD-L1 expression in cervical cancer was reported in several studies [35,36,37].

Pembrolizumab, a IgG4 antibody targeting the PD-1 receptor in the cell surface, was studied in the Keynote 826 phase 3 trial on patients receiving first-line chemotherapy for persistent, recurrent, or metastatic cervical cancer. The patients assigned to receive pembrolizumab had improvement in median progression-free survival (PFS) compared to those who received placebo (10.4 versus 8.2 months; hazard ratio (HR) for disease progression or death 0.65, 95% confidence interval (CI) 0.53–0.79). Overall survival (OS) was 50 percent in the pembrolizumab group and 40 percent in the placebo group at two years (HR 0.67, 95% CI 0.54–0.84). In addition, the objective response rates were 66 and 51 percent, respectively [38]. Pembrolizumab was approved for subsets of patients with advanced cervical cancer that has PD-L1 expression, high microsatellite instability (MSI-H), or high tumor mutational burden (TMB-H).

Another PD-1 inhibitor, Nivolumab, was studied in the CheckMate 358 study. The study was a phase 1/2 clinical trial investigating nivolumab for virus-associated tumors, including 18 cervical cancer patients. Promisingly, the overall response rate was 26.3%. In addition, a median OS of 21.9 months was demonstrated in the entire cohort [39].

Vascular endothelial growth factor (VEGF) and epidermal growth factor receptor (EGFR) can also be targeted for monoclonal antibody (mAb) specific treatment in cervical cancers. Apart from HPV being the causative agent in cervical cancer, the deactivation of wild-type p53 was found to have an effect in the formation of new blood vessels by enhancing the expression of VEGF, which can have a profound impact on cancer progression, treatment response, and the outcome in patients [40,41,42,43,44]. Hence, it is apparent from the previous findings [40,45,46] that VEGF, known for its primary role in neo-angiogenesis, showed very high expression levels during tumor development in the cervical region; consequently, it has drawn research interest for mAb immunotherapy. Bevacizumab has been in extensive clinical evaluation as an immunotherapeutic drug, either as individual mAb or in combination against cervical cancer after radiation therapy and one chemotherapy regimen. Results indicate that a few of the clinical patients benefitted, with no tumor growth/recurrence for over six months, and a few have shown partial response towards the bevacizumab treatment [47,48,49]. For women with recurrent, metastatic, or advanced cervical cancer, platinum-based chemotherapy and the angiogenesis inhibitor bevacizumab are recommended. This recommendation is based on the results of the Gynecologic Oncology Group (GOG) protocol 240, in which 452 women with metastatic, persistent, or recurrent cervical carcinoma were randomly assigned to chemotherapy with or without bevacizumab. Chemotherapy plus bevacizumab resulted in a significant improvement in overall survival and progression-free survival compared with chemotherapy alone (median OS, 16.8 versus 13.3 months, respectively; HR 0.77, 98% CI 0.62–0.95 and median PFS, 8.2 versus 6.0 months; HR 0.68, 95% CI 0.54–0.82) [50]. These results support the use of chemotherapy plus bevacizumab as a first-line treatment of metastatic cervical cancer. In 2014, the United States FDA approved bevacizumab for this indication. The recent developments and strategies for the immunotherapy of cervical cancer has been extensively discussed elsewhere [51,52,53,54]. In addition, other checkpoint inhibitors are being investigated and should offer more alternative options for cervical cancer treatment in the future. However, the cost of therapy is a challenging barrier, most notably in LMIC.

## 5. Plant Platform for the Production of Affordable Biopharmaceuticals

Over the past two decades, several mAbs and recombinant vaccines have been approved by the FDA for treatment of various malignancies, revolutionizing cancer treatment. Different expression systems are employed for the production of recombinant vaccines and mAbs, including yeast, insect cells, and mammalian cells [55]. Most of the currently available vaccines or drugs are expensive due to the complex manufacturing processes, including the upstream scale-up and downstream purification associated with existing platforms. It is worth noting that plants are employed for the large-scale production of vaccines, mAbs, and diagnostic reagents for several infectious diseases, including, but not limited to, the Coronavirus disease (COVID-19), human immunodeficiency virus (HIV), and influenza [56,57,58,59,60], which has encouraged the industrial adoption of this platform in recent years. Nonetheless, the striking features of the plant platform—such as low costs, convenient upstream and downstream steps for protein production, along with product safety and efficacy—makes the plant-based platform suitable for the needs of developing world markets. Recent advancements in plant transient expression and glycoengineering open an avenue for the economical, large-scale production of vaccine antigens, monoclonal antibodies, and other biologics for cancer treatment. In the next six years, the market size of global plant-based vaccines is predicted to grow over 11.7%, compared to the estimated value of USD 927 million in 2020, which indicates that the plant vaccine production sector is one of the markets with the most potential [61]. The platform has grown consistently over the last three decades, mainly due to new technological advances in recombinant protein production. It is important to note that plant-based vaccines against several diseases have showed better safety and efficacy, even in clinical trials [59,62,63,64].

Plant-based vaccine production mainly relies on the transformation of a target gene sequence cloned in the expression vector into plant cells. For large-scale production, gene encoding either vaccine antigen or light chain and heavy chain of the mAb cloned in the plant expression vector were transformed into plants, either for stable or transient expression. In stable expression, the desired gene sequence is integrated into the DNA of the nucleus or chloroplast in the host cell, thereby altering the host genome. The biolistic method (particle bombardment) or *Agrobacterium tumefaciens* can be utilized to generate the genetically modified plants. The transformed plants are cultivated *in vitro*, allowing the regeneration of transgenic/transplastomic plants, or the transformed plant cells were propagated in liquid medium, which can be used as a cell-suspension culture platform. However, the yield of the recombinant protein was less and the process was also time-consuming.

The transient expression approach based on *A. tumefaciens* (Agroinfiltration) and/or viral vector-mediated protein expression is rapid, and large quantities of antigen or protein of interest can be produced within 6–8 weeks of the delivery of the desired gene sequence. Syringe or vacuum infiltration can be employed to introduce the *A. tumefaciens* suspension into the plant cells. Unlike stable transformation, the foreign gene sequences in the expression vector are not integrated into the DNA of the host cells in transient expression [65].

## 6. Plant-Made Biopharmaceuticals as Anticancer Therapeutics

### HPV Vaccines

The causative agent of cervical cancer is HPV. Thus far, several vaccine candidates against HPV have been expressed in plants and characterized in animal models (Table 2). In recent years, the plant-based platform has been applied for the production of cancer vaccines, mAbs, and related biologics. Few plant-made vaccine prototypes have been reported and characterized for cervical cancer. Several plant expression approaches—including stable transformation (nucleus and chloroplast) and transient methods (Agroinfiltration and viral vector mediated)—were employed for the production of HPV antigens in plants. Mendoza et al. performed an extensive analysis and reviewed the potential of plant-derived vaccines against cancer [66,67]. HPV capsid, L1 protein is the most highly conserved of all papillomavirus proteins. L1 protein self-assembles into VLPs when expressed in heterologous systems [68]. Hence, these VLPs are considered a key candidate for the development of prophylactic vaccines against HPV. Currently, several potential HPV therapeutic vaccines produced in plants enhance tumor regression in animal models and evoke specific cell-mediated immune responses.

A plant-based vaccine against human papillomavirus used transient expression of HPV 16 E7 protein in *N. benthamiana* using a potato virus X-derived vector. The immunogenicity studies in mice showed that the mice immunized with E7-containing foliar extracts induced anti-E7 specific IgGs. Furthermore, anti-E7-specific cell-mediated immune response was confirmed by ELISpot results. The vaccinated mice were protected from tumor progression following challenge with E7-expressing C3 tumor cells. Approximately 40% of the vaccinated animals were protected, and the remaining 60% of the animals showed delayed tumor growth [69]. These studies were further extended to test the prophylactic and therapeutic efficacy of the vaccine candidate against HPV-induced tumors. In this regard, the wild type E7 or the E7GGG mutant fused to *Clostridium thermocellum β*-1,3-1,4-glucanase (LicKM) were transiently expressed in *N. benthamiana* plants. The plant-produced vaccine exerted promising therapeutic and prophylactic effects. The plant-derived LicKM-E7 and LicKM-E7GGG fusion protein induced both humoral and cell mediated immune response in mice superior to those of the bacterial (*E. coli*)-produced counterparts. It is notable that the animals induced significant antibody responses when adjuvant LicKM fusions were present. In ELISPOT, increasing numbers of IFNγ secreting cells were observed in mice immunized with LicKM-E7 or LicKM-E7GGG, whereas *E. coli*-derived E7 or E7GGG did not induce E7-specific CD8+ cells in the immunized mice. The immunized mice were challenged with TC-1 cells and the antitumor activity was investigated. Outstandingly, LicKM-E7 or LicKM-E7GGG plus adjuvant-immunized animals did not develop tumors (100% of animals), whereas *E. coli*-derived E7 or E7GGG with adjuvant protects 80% and 60% of animals, respectively. The therapeutic activity was assessed in mice by inoculating TC-1 tumor cells 3 days prior to LicKM-E7 or LicKM-E7GGG immunization. All animals treated with LicKM-E7 or LicKM-E7GGG plus adjuvant remained tumor free throughout the duration of the study. In contrast, the *E. coli*-produced E7 or E7GGG adjuvanted vaccines inhibited tumors in only 40% and 60% of the animals, respectively. These outcomes provide an insight on the production of both prophylactic and therapeutic anti-tumor vaccine candidates with LicKM fusions in plants [73].

Efforts to develop oral HPV vaccine was demonstrated by Warzecha et al. This pioneering study reported the assembly of human papillomavirus-like particles by expressing HPV type 11 (HPV11) L1 major capsid protein in transgenic potato plants. The production yields of L1 VLPs was ~20 ng/g of fresh weight. Further, ingestion of transgenic L1 potato tubers along with mucosal adjuvant *E. coli* toxin LT(R192G) induced a humoral response in animals, and the response increased significantly when boosted with insect-cell-derived VLPs [80]. As the immune response induced by this vaccine relies on the adjuvant and boosting, the authors concluded that the low immunogenicity could be due to the low expression of L1 VLPs in plants.

A similar study reported by Liu et al. demonstrated the assembly of VLP by expressing the major capsid protein of HPV 16, L1 protein under the constitutive Cauliflower mosaic virus (CaMV) 35S promoter in *N. tabacum L*. cultivar Xanthi plants by *Agrobacterium*-mediated stable transformation. The stable integration of the transgene in the host genome and its expression was confirmed by Southern blot and western blot, respectively. The recombinant protein accumulated up to 0.034%–0.076% of the total soluble leaf protein in the transformed plants [85]. Nevertheless, this study did not test the immunogenic potential of the plant-produced antigen.

In another study, the HPV-16 L1 antigen was expressed in chloroplasts of tobacco plants by stable nuclear expression. Earlier reports on the nuclear transformation of HPV antigens reported low levels of protein expression, whereas in this study, high expression levels in the range of 2.1 to 3.7 mg L1/g fresh weight, which is equivalent to 20–26% of total soluble protein, was reported. Almost ~240 mg of L1 protein has been produced from a single mature plant. The high protein accumulation is attributed to the high transgene copy number or the higher protein stability in the chloroplast. The immunogenicity of chloroplast-derived L1 protein was further tested in mice. Balb/c mice immunized intraperitoneally with L1-transformed plant extract induced anti-L1 antibodies when co-administered with Freund’s adjuvant or aluminium hydroxide as adjuvant after the second booster, whereas no antibodies were detected in the control group immunized with wild-type plant extracts. This study indicates that the chloroplast-derived L1 antigen is immunogenic in mice. The advantages of this study are the higher protein accumulation and the inclusion of approved alum adjuvant in animal experiments, which might facilitate regulatory approval for clinical trials if the vaccine is adopted in further studies. Nonetheless, no challenge experiments were performed to evaluate the significance of humoral response [89].

Another promising report on VLP production has been led by Rosa et al. Chimeric HPV 16 VLPs containing L1 fused to a string of epitopes from HPV 16 E6 and E7 proteins were stably expressed in tomato plants at the level from 0.05% to 0.1% of total soluble protein. The integration of transgene in the host genome was confirmed by southern blotting, and its expression was confirmed by northern and western blotting. Intraperitoneal administration of tomato-derived VLPs in mice induced neutralizing antibodies and cytotoxic T-lymphocytes. Moreover, the mice were protected following the challenge with TC-1 cells. These findings are promising, and this is the first report on chimeric VLP production in plants. In further characterization, mice were immunized intraperitoneally with three doses of 5 μg of VLP, along with Freund’s adjuvant. The antibody titers elicited in plant-produced chimeric VLPs were similar to those of commercial vaccines. Notably, the antibody levels were measured at 6 and 12 months after administration, suggesting a long-lasting immune response induced by the vaccine. Furthermore, a significant reduction of tumor growth was observed in the C57BL/6 mouse model challenged by TC-1 cells. These findings proved that this promising candidate could be further evaluated in safety and efficacy studies so that this candidate can be developed as a broad-spectrum HPV vaccine [92,93].

Using *N. benthamiana*, high levels of shuffled HPV-16 E7 (16E7SH) was produced by fusing with a self-assembly domain of the maize γ-zein (Zera), which is a signal sequence that helps in the formation of protein bodies. The fusion domain promotes the recombinant protein accumulation in the form of protein bodies in the endoplasmic reticulum, protects proteins from proteolytic degradation, and facilitates purification. The Zera-HPV proteins were accumulated in the tobacco plants at levels ranging from 0.1–6 g/kg. The immunogenicity of the plant-produced proteins was assessed in C57BL/6 mice, and the results demonstrated that the vaccine candidate enhanced the cellular immune response, which can cause tumor regression in mice. Furthermore, the authors proved the adjuvant activity of Zera peptide in enhancing the immune response. Hence, this approach utilizing the Zera based protein bodies could be a potential vaccine candidate due to its advantages of low-cost production and purification [76].

In addition, HPV16 L1 VLPs has been expressed in *N. benthamiana* leaves transfected with the magnICON vector system with a chloroplast signal sequence. Higher accumulation was shown for the construct with L1 and a chloroplast transit peptide sequence, whereas low expression was reported for the construct without the peptide sequence. An average of up to 250 mg of L1 protein was accumulated per kg of plant biomass. The 35–55 nm VLPs were successfully assembled inside the plant cells with the maximal protein level of >2.5% of total soluble protein. The immunogenicity of the plant-purified VLPs were determined by intraperitoneally administering 100 µg of VLPs without any adjuvant in C57BL/6J wild-type mice. The animals were immunized three times in two-week intervals. High antibody titers were observed when the sera collected from the plant-made VLPs immunized animals were titrated with the insect cell VLPs. Nevertheless, the neutralization ability of the plant VLP-elicited antibodies was not evaluated against HPV [91].

In continuation of the previous report by Massa et al., this study evaluated the therapeutic activity of E7* in an animal model. The authors report the expression of HPV type 16 E7* protein in *N. benthamiana* by transient expression technology and tomato hairy root cultures by stable transformation. The E7* protein was fused to SAPKQ, a saporin protein from *Saponaria officinalis.* The inclusion of nontoxic saporin protein SAPKQ added an advantage to the vaccine because it can improve cell-mediated responses. The immune response of the plant-produced vaccine was assessed in C57BL/6 mice. The finely ground root tissues resuspended in phosphate-buffered saline pH 7.2 were used for mice experiments. Animals were primed with the E7*-SAPKQ DNA vaccine (50 μg/mouse) and boosted either with the same DNA or with the E7*-SAPKQ root extract-based vaccine (1 μg/mouse). Alternatively, one group also received two doses of E7*-SAPKQ root extract-based vaccine (prime and boost; homologous schedule) with 1-week interval. A dramatic increase in cell-mediated immune response was observed in the heterologous prime and boost combinations compared to other combinations. Further, the therapeutic potential was also demonstrated by injecting naïve C57BL/6 mice with 5 × 10^4^ TC-1 tumor cells in saline solution. Three days after the challenge, mice were immunized with the standard vaccine preparations following the same regimen as the immunization protocol. The heterologous schedule showed significant differences with respect to homologous schedules. This is the first report that showed the therapeutic activity of a hairy root-derived HPV candidate in animal studies [79].

In a recent study, Naupu et al. reported the protective effect of L1 proteins of eight high-risk (HPV 16, 18, 31, 33, 35, 45, 52, and 58) and two low risk (HPV 6 and 34) HPV types produced in *N. benthamiana* by transient expression. The immunogenicity of the plant-produced antigen of three oncogenic HPV types HPV 35, 52, and 58 was assessed by subcutaneous immunization of BALB/c mice using the Montanide ISA 50 V2 adjuvant every two weeks on day 3, 17, 31, and 45, and the blood was collected on day 56. The animals were boosted on day 59, and the final bleed was obtained on day 73. Gardasil, an approved HPV vaccine, was used as a positive control. The vaccine-immunized mice significantly induced anti-L1 immune responses, compared to the negative control group, as well as type-specific neutralizing antibodies that can neutralize homologous HPV pseudovirions *in vitro*, whereas the plant-produced serum did not neutralize heterologous pseudovirions. The immune response induced by the plant-produced vaccines cannot be compared directly with the approved vaccine Gardasil due to the fact that both the vaccines were produced on different platforms, formulated with different adjuvants, and administered in different concentrations/doses. However, the plant-derived vaccine elicited a comparable response to that induced by the commercial Gardasil vaccine, suggesting the potential of the plant platform to produce cost-effective HPV vaccine [100].

The positive outcomes of a number of HPV candidates expressed in plants reflects the realistic potential for the development of plant-based vaccines against HPV. Looking at a larger scale, plant transient expression in *Nicotiana* spp. is a promising option to produce multivalent HPV vaccines by expressing VLPs from distinct virus types and formulating thereafter. It is envisaged that the implementation of improved expression strategies to increase protein yields in edible crop plants represents an alternative strategy. Further, the exploration of novel vaccines consisting of chimeric protein with immunogenic epitopes from different HPV could offer broader immunoprotection against several HPV types. Another promising strategy is designing multi-epitope or peptide vaccines utilizing immunoinformatic tools and computational approaches that are capable of stimulating both humoral and cellular immunity. Thus far, the preliminary studies performed with the HPV vaccine candidates produced in plants proved that the vaccine is immunogenic and inhibits tumor growth in animals. Furthermore, evaluating the efficacy, safety, and toxicity at the preclinical level is the next big step to push forward plant-made cancer vaccines to clinical applications.

## 7. Conclusions and Future Prospects

Cancer immunotherapy has become the versatile and non-invasive approach in cancer therapy for treating several human cancers, along with chemotherapy, radiation therapy, or other immunomodulators. Therapeutic antibodies target immune checkpoint molecules on immune cells or tumor cells and can also exert enhanced anti-tumor effects by manipulating tumor-related signaling. In addition, targeting the pathogens that contribute to cancer development by utilizing vaccination strategies could also reduce risk. However, access to life-saving medicines or drugs is challenging given current market prices and global production capacities based on traditional bacterial or mammalian cell-based systems. Due to the limited availability of preventive vaccines against HPV infections in developing countries, it is highly unlikely to control HPV infections in those places, and it could take years to make a positive impact on reducing the cancer. The induction of neutralizing antibody responses against HPV can prevent HPV infection by blocking its entry into host cells. The demand for therapeutic proteins for cancer immunotherapy, in which plants have the capability to produce enough biopharmaceuticals to cope with demand, is increasing every year. The current scenario of plant-based vaccines targeting cancer is the result of coordinating efforts among many scientific groups worldwide. There are enormous success stories on the aspect of production of functional plant-derived antigens and mAbs in recent decades. Plants are a promising platform for the industrial-scale manufacturing of recombinant proteins due to the low cost and high scalability in short time. Further, several challenges associated with upstream and downstream manufacturing of recombinant therapeutic proteins in plants, such as low expression, yield, and purity of final products, has been addressed. The advances in plant transient expression and glycoengineering technologies has made plants a real competitor to the traditional mammalian-based system. Further, the regulatory frameworks for plant-derived biopharmaceuticals are becoming well defined and the technology has finally come of age. However, co-ordination between academic organizations and biopharmaceutical companies or government agencies in developing countries is essential for capacity building and to accelerate the development of plant-based biomanufacturing facilities locally.

In conclusion, cancer treatment could be effective and yield the desired effect if immunotherapy methods are combined with traditional therapies or other targeted methods in order to overcome the limitations of any particular treatment method. Hence, in subsequent clinical trials, it is vital to determine the suitable combination of methods that can give the best results. Further, plants are a promising platform to produce cost-effective, efficacious vaccines against HPV that could greatly benefit public health due to their low cost. Additionally, plant production systems can self-assemble HPV VLPs, which emphasizes its role as a suitable platform for production of VLP-based vaccines. Preclinical and clinical evidence of a few plant-produced pharmaceuticals against other viral infections will pave the way to develop plant-derived vaccines and mAbs for cancer treatment that could eventually reduce the financial burden, particularly in resource-limited settings. The progress of the approval of the first plant-based biopharmaceuticals, ZMapp by the FDA and Medicago’s Covifenz COVID-19 vaccine by Health Canada, has displayed the potential of the plant platform for vaccine and mAb production. It is envisaged that the plants can be used for the production of low-cost, efficacious HPV vaccines, which will greatly improve the outcome and quality of the patient’s life, particularly in LMICs. Nevertheless, plant-based biologics have a great potential for enhancing human health, which requires the demonstration of product efficacy, safety, and feasibility for making a significant impact on the market. Still, extensive research is needed to shift the proof-of-concept findings of plant-made cancer vaccines to clinical applications, which is a major step to bring plant-derived candidates to the market.

## Figures and Tables

**Table 1:** Comparison of HPV vaccines that are approved for clinical use.

HPV Vaccines			
Trade Name	**Gardasil**	**Cervarix**	**Gardasil 9**
Approval	2006	2009	2014
Manufacturer	Merck & Co	GlaxoSmithKline	Merck & Co
HPV types	6, 11, 16, and 18	16 and 18	6, 11, 16, 18, 31, 33, 45, 52, 58
Expression system	Yeast (*Saccharomyces cerevisiae* CANADE 3C-5 (Strain 1895)	Baculovirus expression system (Hi-5 Rix4446 cells derived from *Trichoplusia ni*)	Yeast (*Saccharomyces cerevisiae* CANADE 3C-5 (Strain 1895)
Adjuvant	Aluminium hydroxyphosphate sulfate	MPL and aluminum hydroxide (AS04)	Aluminium hydroxyphosphate sulfate
Excipients	Sodium chloride, Histidine, Polysorbate 80, Borax, Water for injections	Sodium chloride, sodium dihydrogen phosphate dihydrate, water for injections	Sodium chloride, Histidine, Polysorbate 80, Borax, Water for injections
Dose	0.5 mL/dose	0.5 mL/dose	0.5 mL/dose
Administration schedule	0, 2 and 6 months	0, 1 and 6 months	0, 2 and 6 months
Routes of administration	Intramuscular injection deltoid area of the upper arm or in the higher anterolateral area of the thigh	Intramuscular injection in the deltoid region	Intramuscular injection in the deltoid or anterolateral area of the thigh
Storage	2 °C to 8 °C	2 °C to 8 °C	2 °C to 8 °C
Efficacy	70–75%	70%	90%

**Table 2:** List of vaccine candidates expressed in plants against HPV.

Antigen	Plant System	Transformation Method	Expression Level	Immunization Route	Outcome	Reference
HPV-16 E7 protein	*Nicotiana benthamiana* (Tobacco)	Plant virus infection (Transient expression)	3–4 μg/g fresh weight	Subcutaneously injected in mice	E7-specific CD8^+^ cytotoxic T cells were stimulated in mice; Both humoral and cell-mediated responses were induced; about 40% of mice were protected after C3 tumor cells challenge	[69]
	*Nicotiana benthamiana* (Tobacco)	Plant virus infection (Transient expression)	15 μg/g of fresh weight	Subcutaneously injected in mice	Strong cell-mediated immune response was induced; Increased tumor protection in about 80% of mice after tumor challenge	[70]
	*Nicotiana benthamiana* (Tobacco)	Plant virus infection (Transient expression)	NA	NA	Dendritic cells pulsed with plant extract containing E7 were able to prime human blood-derived lymphocytes from healthy individuals to induce HPV16 E7-specific cytotoxic response	[71]
	*Nicotiana tabacum* cv. Petit Havana (Tobacco)	Biolistic method (Transplastomic expression/Chloroplast)	0.1% total soluble protein	NA	NA	[72]
HPV-16 E7CP protein	*Nicotiana tabacum* cv. Petit Havana (Tobacco)	Biolistic method (Transplastomic expression/Chloroplast)	0.5% total soluble protein	NA	NA	[72]
HPV-16 LicKM-E7GGG fusion protein	*Nicotiana benthamiana* (Tobacco)	Plant virus infection (TMV) (Transient expression)	400 mg/kg fresh weight	Subcutaneously injected in mice	Strong humoral and cell-mediated immune responses was induced in mice; Tumor protection in 100% of animals after tumor challenge	[73]
	*Nicotiana benthamiana* (Tobacco)	Plant virus infection (TMV) (Transient expression)	NA	Subcutaneously injected in mice	Inhibition of tumor growth in vaccinated mice after challenge; Increased overall survival of treated mice	[74]
	*Nicotiana benthamiana* (Tobacco)	*Agrobacterium* mediated (Transient expression)	233 mg/kg fresh weight	NA	NA	[75]
HPV-16 E7-Zera	*Nicotiana benthamiana* (Tobacco)	*Agrobacterium* mediated (Transient expression)	150 mg/kg	NA	NA	[76]
HPV-16 E7SH-Zera fusion protein	*Nicotiana benthamiana* (Tobacco)	*Agrobacterium* mediated (Transient expression)	1100 mg/kg	Subcutaneously injected in mice	Both humoral and potent cell-mediated immune responses are induced; tumor regression in mice were elicited	
HPV-16 LALF_32–51_-E7 fusion protein	*Nicotiana benthamiana* (Tobacco)	*Agrobacterium* mediated (Transient expression)	Up to 0.5% total soluble protein	NA	NA	[77]
	*Nicotiana benthamiana* (Tobacco)	*Agrobacterium* mediated (Transient expression)	NA	NA	NA	[78]
HPV-16 E7 *-SAPKQ fusion protein	*Solanum lycopersicum* cv. Micro-Tom (Tomato)	*Agrobacterium* mediated (Hairy root cultures)	35.5 μg/g fresh weight	Subcutaneously injected in mice	Hairy root extract as boost induced cell-mediated immune response and demonstrated anticancer activities against HPV TC-1 tumor cells	[79]
HPV-11 L1 capsid protein	*Solanum tuberosum* cv. Desiree (Potato)	*Agrobacterium* mediated (Transgenic expression/Nucleus)	20 ng/g fresh tuber	Oral ingestion (Feeding of tubers in mice)	Anti-L1 immune response was activated and enhanced after subsequent boosting with insect cell derived VLP	[80]
	*Arabidopsis thaliana* ecotype Columbia (Thale cress)	*Agrobacterium* mediated (Transgenic expression/Nucleus)	Up to 12 μg/g fresh weight	Subcutaneously and intramuscularly injected in rabbits	Weak antibody response and antisera were not reactive with native HPV-11 L1 VLPs and not able to neutralize HPV-11 pseudovirion *in vitro*	[81]
	*Nicotiana tabacum* cv. Xanthi (Tobacco)	*Agrobacterium* mediated (Transgenic expression/Nucleus)	Up to 2 μg/g fresh weight			
CRPV L1 capsid protein	*Nicotiana tabacum* cv. Xanthi (Tobacco)	*Agrobacterium* mediated (Transgenic expression/Nucleus)	Up to 1.0 mg/kg leaf weight	Subcutaneously and intramuscularly injected in rabbits	L1-specific antibodies were elicited from both transiently and transgenically produced CRPV L1 but stronger to those immunized with TMV-derived L1 protein. No neutralization of pseudovirus was observed *in vitro*; Rabbits were protected against wart development after CPRV challenge	[82]
	*Nicotiana benthamiana* (Tobacco)	Plant virus infection (TMV) (Transient expression)	Up to 0.4 mg/kg leaf weight			
HPV-16 L1 capsid protein	*Nicotiana tabacum* cv. Samsun NN (Tobacco)	*Agrobacterium* mediated (Transgenic expression/Nucleus)	0.5% total soluble protein	Subcutaneously injected in mice	HPV-16 L1-specific antibodies were elicited, and titers were equal to those produced by immunization using insect cell-derived VLP	[83]
	*Solanum tuberosum* cv. Solara (Potato)	*Agrobacterium* mediated (Transgenic expression/Nucleus)	0.2% total soluble protein	Oral ingestion (Feeding of tubers in mice)	Weak but detectable anti-L1 antibody response	
	*Nicotiana tabacum* cv. Xanthi (Tobacco)	*Agrobacterium* mediated (Transgenic expression/Nucleus)	2–4 µg/kg fresh weight	Subcutaneously and intramuscularly injected in rabbits	No adverse effects observed in immunized rabbits; weak anti- L1 immune response elicited with low doses	[84]
	*Nicotiana tabacum* cv. Xanthi (Tobacco)	*Agrobacterium* mediated (Transgenic expression/Nucleus)	0.034–0.076% total soluble protein	NA	Plant-derived L1 induced murine erythrocyte hemagglutination *in vitro*	[85]
	*Nicotiana benthamiana* (Tobacco)	Plant virus infection (TMV) (Transient expression)	20–37 μg/kg fresh weight	Subcutaneously and intramuscularly injected in rabbits	Weak L1-reactive antibodies were elicited	[86]
	*Nicotiana tabacum* L. ‘Petit Havana’ SR1 (Tobacco)	*Agrobacterium* mediated (Transient expression)	40 mg/kg fresh weight	Subcutaneously injected in mice	High titres of HPV-16 L1-specific antibodies and strongly neutralizing antibodies were elicited	[87]
	*Nicotiana benthamiana* (Tobacco)	*Agrobacterium* mediated (Transient expression)	>17% total soluble protein	NA	NA	
	*Nicotiana tabacum* L. ‘Petite Havana’ SR1 (Tobacco)	*Agrobacterium* mediated (Transgenic expression/Nucleus)	Up to 11% total soluble protein	NA	NA	
	*Nicotiana tabacum* cv. Petit Havana (Tobacco)	Biolistic method (Transplastomic expression/Chloroplast)	1.5% total soluble protein	NA	NA	[88]
	*Nicotiana tabacum* L. Petite Havana (Tobacco)	Biolistic method (Transplastomic expression/Chloroplast)	24% total soluble protein	Intraperitoneally injected in mice	HPV-16 L1-VLPs are highly immunogenic and neutralizing antibodies were detected in sera from immunized mice	[89]
	*Nicotiana benthamiana* (Tobacco)	*Agrobacterium* mediated (Transient expression)	Up to 550 mg/kg fresh weight	NA	NA	[90]
	*Nicotiana benthamiana* (Tobacco)	*Agrobacterium* mediated (Transient expression)	>2.5% total soluble protein	Intraperitoneally injected in mice	High reactivity and antibody titers were detected from immunized sera	[91]
HPV-16 L1 and L1-E6/E7 chimera	*Lycopersicon esculentum* Mill. (Tomato)	*Agrobacterium* mediated (Transgenic expression/Nucleus)	0.05–0.1% total soluble protein	Intraperitoneally injected in mice	Neutralizing antibodies and cytotoxic T cell activity to L1, E6/E7 were elicited	[92]
	*Lycopersicon esculentum* (Tomato)	*Agrobacterium* mediated (Transgenic expression/Nucleus)	NA	Intraperitoneally injected in mice	Reactive and neutralizing IgG antibodies were persistent for over 12 months; Significant reduction in tumor growth (57%) in treated mice was observed	[93]
HPV-16 L2-ACP-E7 fusion protein	*Nicotiana benthamiana* (Tobacco)	*Agrobacterium* mediated (Transient expression)	Up to 0.043 mg/g fresh weight	NA	NA	[94]
Mutated HPV-16 L1 (L1_2xCysM)	*Nicotiana tabacum* cv. Petit Havana (Tobacco)	Biolistic method (Transplastomic expression/Chloroplast)	Up to 1.5% of total soluble protein	NA	NA	[95]
HPV-16 L1 (L1ΔC22 and chimaeras bearing M2e influenza epitope)	*Nicotiana benthamiana* (Tobacco)	*Agrobacterium* mediated (Transient expression)	Up to 3.9% total soluble protein	NA	NA	[96]
HPV-16 L2- Potato virus X coat protein (PVX CP) fusion protein (N-Terminal)	*Nicotiana benthamiana* (Tobacco)	*Agrobacterium* mediated (Transgenic expression/Nucleus)	170 mg/kg fresh weight	Subcutaneously injected in mice or administered into skin by a tattoo device	PVX CP and L2-specific antibodies were elicited in mice sera	[97]
HPV-16 PVX CP-L2 fusion protein (C-terminal)	*Nicotiana benthamiana* (Tobacco)	*Agrobacterium* mediated (Transgenic expression/Nucleus)	8 mg/kg fresh weight	NA	NA	
HPV-16 L1/L2 chimeras	*Nicotiana benthamiana* (Tobacco)	*Agrobacterium* mediated (Transient expression)	~1.2 g/kg plant tissue	Subcutaneously injected in mice	L1/L2 (108–120) elicited anti-L1 and anti-L2 antibody responses which were able to neutralize homologous HPV-16 and heterologous HPV-52 pseudovirions	[98]
	*Nicotiana benthamiana* (Tobacco)	*Agrobacterium* mediated (Transient expression)	Up to 145 mg/kg fresh weight	Subcutaneously injected in mice	Cross-neutralization for other HPV types (HPV-11, -18, and -58) with antisera specific to chimeras; L1-specific antibodies can neutralize homologous HPV-16 (anti- SAE 65–81 antiserum)	[99]
HPV L1 capsid protein	*Nicotiana benthamiana* (Tobacco)	*Agrobacterium* mediated (Transient expression)	NA	Subcutaneously injected in mice	L1-specific antibodies were produced which were able to successfully neutralize homologous HPV pseudovirions in pseudovirion-based neutralization assays	[100]

Note: NA—not applicable or information not available.

## Data Availability

Not applicable.
